# Spore Evidence for the Origin of Isoetalean Lycopsids?

**DOI:** 10.3390/life13071546

**Published:** 2023-07-12

**Authors:** Jiří Bek, Jana Votočková Frojdová

**Affiliations:** Institute of Geology, Academy of Sciences of the Czech Republic, Rozvojová 269, 16500 Prague, Czech Republic; frojdova@gli.cas.cz

**Keywords:** lycopsids, isoetaleans, selaginellaleans, rhyniophytes, spores, palynology, Paleozoic

## Abstract

A new hypothesis about the origin of isoetalean lycopsids was proposed based on palynological data. The occurrence of three apical papillae on the proximal surfaces of miospores is a significant palynological feature that is clearly defined in both isoetalean and selaginellalean clades. Three apical papillae appeared for the first time within lower Silurian (Wenlockian ca. 430 My) and only in rhyniophytoid plants. Using this observation, we suggest that isoetalean lycopsids could have evolved directly from rhyniophytoids and not from protolepidodendralean lycopsids in the middle Devonian (Eifelian–Givetian) as previously suggested, because protolepidodendralean spores do not possess three apical papillae. Spores with three apical papillae, reported as dispersed as well as in situ, were recorded continuously from the lower Silurian (Wenlockian) through the Devonian, Carboniferous, Permian, Mesozoic to Cenozoic era and form a phylogenetically independent lineage.

## 1. Introduction

Lycopsids are a monophyletic group of extant plants with about 1250 species growing globally in all climatic belts [1] and are believed to be the oldest living lineage of vascular plants. It is possible to state that the “golden age” for lycopsids was the Carboniferous era (358.9–298.9 My) especially in coal-forming swamps in tropical wet areas where lycopsids were the dominant plant group and the most important contributors to biomass. The position of sporangium in the axil or on the upper surface of the leaf or sporophyll is one of the most important features. Sporophylls can be among photosynthetic microphylls or (as non-photosynthetic sporophylls) aggregated into cones or fertile zones [2]. The sporangia are on the adaxial side, i.e., upper side of the leaf. Sporophytes are the dominant form. The majority of lycopsids produce one type of spores (homosporous) but some forms yield two types (heterosporous). Some cones possess only one type (monosporangiate) while others have two types of spores (bisporangiate). Zosterophylls were probably a sister group to lycophytes [1]. Today lycopsids are herbaceous but, especially during the Carboniferous, many of them were arborescent in form. Three lycopsid orders, i.e., Lycopodiales, Selaginellales and Isoetales are usually recognized as three independent lineages/clades [1].

### 1.1. Isoetalean Clade

The isoetalean clade is divided into the orders Lepidodendrales and Isoetales. Lepidodendrales were arborescent forms up to 45 m high [3] with typical stigmarioid rhizomorphs. The most abundant were the genera *Lepidodendron*, *Lepidophloios*, *Diaphorodendron, Paralycopodites* and *Sigillaria*. The Lepidodendralean clade can be subdivided into Monosporangiate-strobilus (*Lepidostrobus*, *Sigillariostrobus*) and Bisporangiate-strobilus (e.g., *Flemingites*, *Thomasostrobus*) subclades [4]. Palynologically Carboniferous lepidodendralean spores are represented mainly by the genera *Lycospora, Densosporites, Crassispora, Cirratriradites* and *Endosporites* and several of them have palaeoecological importance [1]. It was supposed [1] that isoetalean lycopsids originated from protolepidodendraleans, with the first lepidodendraleans appearing probably within the late Devonian [1]. Today only about 150 cosmopolitan species with elongated leaves that are aquatic to semi-aquatic are known [1]. The dominant plant habit of modern isoetes, a reduce cormose form that lacks appreciable stem elongation, originated at least by the Jurassic and typifies late Mesozoic and Cenozoic isoetaleans [1]. Post-Paleozoic genera include mainly *Pleuromeia*, *Annalepis*, *Viatscheslavia* and *Tomiotrobus*.

### 1.2. Selaginellalean Clade

Paleozoic selaginellas were different from arborescent lepidodendralean forms because they were small herbs and formed the ground cover in open habitats or the floor layer of the more closed forest communities. The best known are the genera *Selaginella* and *Selaginellites*; the less common ones include *Paurodendron, Carinostrobus, Porostrobus, Bothrodendrostrobus* and *Thomasites*. Palynologically Paleozoic selaginellalean miospores include the genera *Anapiculatisporites, Thomasospora, Cirratriradites, Densosporites* and *Cingulizonates*.

The stratigraphical position of the first undisputed selaginellalean herbs is still far from certain. The Silurian taxa *Baragwanathia*, *Drepanophycus* and *Asteroxylon* are sometimes mentioned among the first possible selaginellalean lycopsids [5]. Silurian taxa were isosporous whereas Carboniferous were bisporangiate. Many Paleozoic herbaceous lycopsids are very similar to the extant genus *Selaginella* which is the plant genus with the longest stratigraphical history from the Paleozoic to recent times. Today, we know about 700 extinct species of selaginellalean plants [1].

### 1.3. Lycopodialean Clade

Lycopodiales originated within the Wenlockian–Přídolian interval [1]. Three lineages evolved during the Lower Devonian: zosterophylls (e.g., genera *Zosterophyllum, Sawdonia* and *Gosslingia*), Asteroxylales (e.g., *Asteroxylon*) and Drepanophycales (e.g., *Drepanophycus*). Extinct genera include *Lycopodium, Lycopodiella* and *Phylloglossum.* Lycopodialean plants are isosporous with apical strobili. An important genus is *Lycopodites* within the Paleozoic as well as extant taxa. Today, Lycopodiaceae has 14 cosmopolitan genera with more than 400 species [6] and they are 5–20 cm tall. The microphylls often densely cover the stem in a linear, scale-like or adpressed fashion to the stem. The leaves are either oppositely or spirally arranged. Paleozoic lycopodialean spores include those from the genera *Retusotriletes* and *Apiculiretusispora*.

### 1.4. Protolepidodendrales

Protolepidodendralean plants were mostly herbs, but sometimes subwoody and small trees that occurred from the Devonian to lower Mississippian. Unlike all other lycophytes however, the Protolepidodendrales bore leaves which were forked at the tips. Protolepidodendralean plants were the first members of the lycophyte lineage to evolve wood and bark, a modified shoot system that acts as a rooting system, bipolar growth and an upright habit [1]. Almost all of them were aligulate [2]. This group underwent a dynamic development during the last 25 years because more than a half of protolepidodendralean taxa have been erected during the last quarter of century, most of them from the late Devonian of China [7]. The most common protolepidodendralean plant genera are *Barsostrobus, Hefengiostrobus, Cyclostigma, Hoxtolgaya, Leclercquia* and *Minarodendron.* Protolepidodendralean lycopsids produced micro- and megaspores [7] but none of their spores possessed three apical papillae. Protolepidodendralean miospores are represented mainly by those of the genus *Acinosporites* and megaspores by those of the genera *Lagenicula* and *Lagenoisporites.* Protolepidodendraleans had an important position in phylogenetical scheme [1] because they should be a parent group for the subsequent origin of the selaginellalean and isoetalean clades.

### 1.5. Rhyniopsida

The Rhyniopsida are a very important group of polysporangiate fossil plants that were defined as plants with naked (without emergences) dichotomizing axes with sporangia that are terminal, mainly fusiform and may dehisce longitudinally; they are diminutive plants and, in so far as is known, have a small terete xylem strand with a central protoxylem [1]. The whole group of rhyniophytoid plants is probably heterogeneous. The oldest unquestionable sporophyte of the earliest vascular land plant is *Cooksonia barrandei* from the Wenlock (432 Ma) Series in the Czech Republic [8] that yielded trilete crassitate spores of the *Aneurospora* type. The position of rhyniopsids is crucial for subsequent plant phylogeny (Table 1). The best-known genus is *Cooksonia* with several fertile specimens that yielded in situ spores [9]. Generally, it is possible to recognize two main morphological types of rhyniopsid in situ spores: crassitate (genera *Ambitisporites, Aneurospora, Streelispora, Synorisporites* and *Retusotriletes*) and non-crassitate (the genus *Apiculiretusispora*). Rhyniophytoid genera that yielded in situ spores include *Cooksonia, Aberlemnia, Concavatheca, Pertonella* and *Renalia*. It is possible to divide rhyniopsida into cooksonioid and renalioid taxa [1]. The genus *Rhynia* gave the name to the whole group and occurs in the *Rhynia* cherts, i.e., the *Rhynia* locality (407 Ma), UK, that represents unique fossilization with animals, fungi, algae and bacteria including the earliest records of plant life cycles.

## 2. Material and Methods

The maceration methods used for dispersed and in situ spores mentioned in this paper were described in the papers of cited authors. Typically, hydrochloric acid was used for 5–24 h, hydrofluoric acid for 3–7 days, hydrochloric acid again for couple of minutes or a few hours. In situ microspores isolated from *Thomasites serratus* were recovered by dissolving small portions of sporangia with the aid of nitric acid (HNO_3_, 60%) for 24–40 h and potassium hydroxide (KOH, 10%). Palynological slides using glycerin jelly for light microscopy observations were made, and some samples were coated with gold for observation using scanning electron microscopy (SEM). The preparation of samples for the study using transmission electron microscopy (TEM) was more complicated and was also described in the papers of cited authors.

Specimens of the Paleozoic selaginellalean species *Thomasites serratus* (specimens Nos. F236113 and F 23629) were from the Ovčín locality, Radnice Basin, Upper Duckmantian of the Czech Republic and the specimens are stored in the paleontological collection of the West Bohemian Museum, Pilsen, Czech Republic. Palynological slides with in situ spores of the *Thomasospora gigantea* type are stored in the Laboratory of Palaeobiology and Palaeoecolgy, Institute of Geology of the Academy of Sciences of the Czech Republic, Prague, and in the collection of the Czech Geological Survey, Prague, Czech Republic. Microspores were examined with a CAMECA SX100 (Laboratory of Palaeobiology and Palaeoecology, Institute of Geology of the Academy of Sciences of the Czech Republic, Prague, Czech Republic) and Tescan Mira3 GMU FEG-SEM (Czech Geological Survey, Prague, Czech Republic) SEM. Photomicrographs were acquired with an Olympus C330s digital camera attached to an OLYMPUS BX51 microscope. Palynological slides with in situ microspores of the *Endosporites globiformis* types isolated from the oldest sub-arborescent isoetalean lycopsid *Polysporia* sp. are stored in the Laboratory of Palaeobiology and Palaeoecology, Institute of Geology of the Academy of Sciences of the Czech Republic, Prague, Czech Republic. Specimens of *Polysporia* sp. (Nos. P-1717/P-1718) are stored in the Cleveland Museum of Natural History, Cleveland, Ohio, USA, and came from the Upper Devonian (Famennian) in the vicinity of Standardsburg in Huron County, Ohio, USA (the Ohio Black Shale).

The methods for chemical and mechanical preparation of spores for TEM are mentioned in the cited papers but usually involved a few weeks of fixation within a 4% paraformaldehyde solution in a phosphate–sodium buffer; the specimens were embedded in agar-agar, washed in distilled water, and then postfixed in a 1% osmium tetroxide solution in phosphate–sodium buffer for 24 h. The samples were dehydrated in a graded ethanol series for 48 h, then immersed in pure propylene oxide and then in mixtures of propylene oxide and an increasing percentage of Epon resin for 24 h. After transfer to pure Epon resin for 24 h, the samples were embedded in fresh Epon resin using flat molds and placed in a 607 °C oven for 48 h for polymerization. The blocks of resin were stored in a closed box containing silica gel. The resin blocks were trimmed and sectioned with a Reichert Ultracut S ultramicrotome using a diamond knife. Sections were collected on uncoated 300-mesh copper grids and stained with a methanol solution of 7% uranyl acetate for 15 min and an aqueous lead citrate solution for 20 min.

## 3. Results

### 3.1. Apical Papillae/Laminated Zones (TAP/LZ)

**Figure 1:**
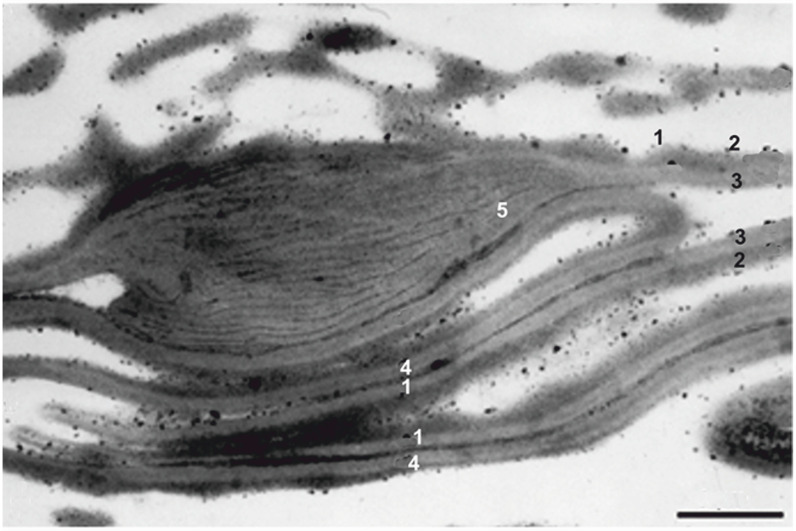
TEM cross section of microspore isolated from *Lepacyclotes* (*Annalepis*) *zeilerii* and compared to the dispersed miospore species *Aratriradites saturni*. 1. Proximal part of exine. 2. Outer exospore. 3. Inner exospore. 4. Distal part of exine. 5. Laminated zones. Scale bar 1 µm. Modified from [23].

The sculpture of the proximal surfaces of spores is variable. It is usually laevigate or variously sculptured (granulate, verrucate, spinate, reticulate). Proximal sculpture elements can be of different types [15], sizes, positions and numbers. Very rarely, some spore taxa possess special proximal sculpture elements called three apical papillae (TAP) [16,17,18,19,20]. TAP are usually three verrucae/granae close to the proximal pole among two rays of the trilete mark. Sometimes they are positive sculpture elements, i.e., elevated above the proximal surface of the spore, and can sometimes be observed only in LM or TEM, i.e., they need not occur on the exine surface but among the exine layers. TAP when studied using TEM are called laminated zones (LZs), i.e., it is a proximal structural change of the exospore [18,19,20]. Proximal exospores include three special zones where the inner layer is markedly thickened and tangentially cleft in ten or so lamnae that are irregularly segmented and interlinked, while the outer layer shows some transversal fissures (Figure 1). These LZs are situated between the aperture arms, near the proximal pole. TAP occur very rarely and are reported from only a few spore-producing genera. TAP/LZs are criteria for the reliable recognition of spores of Paleozoic, Mesozoic and Cenozoic plants of the isoetalean clade [18,19,20,21,22,23,24].

### 3.2. Dispersed TAP Spores

Six Silurian/Devonian and four Carboniferous miospore genera with TAP are known. The Silurian/Devonian genera include *Ambitisporites*, *Synorisporites*, *Retusotriletes, Brochotriletes, Acinosporites* and *Endosporites*.

*Ambitisporites tripapillatus* was reported from the Wenlock to Ludlow of the UK; Wenlock to Pragian of Portugal [25] and Libya [26]; Přídolí of the UK, Libya [26] and Sweden [27]; and Pragian to early Emsian of Argentina [28]. Another *Ambitisporites* TAP species is *A. eslae* which was described from the Přídolí of Sweden [1], Algeria [29] and Libya [30]; late Pragian to early Emsian of Argentina [28]; lower Lochkovian of Brazil [31]; lower Lochkovian to lower Pragian of Spain [32,33,34]; Pragian of France [35]; and Pragian to Emsian of Saudi Arabia [36].

*Synorisporites tripapillatus* is another miospore species with TAP that occurs in the Přídolí of Argentina [37], Sweden [27], the UK, France and Algeria [26]; Ludlow of Spain [26]; and Dowtonian of UK [38].

*Synorisporites papillensis* also possessing TAP was reported from the middle Přídolí of Libya [30,36]; lower Lochkovian to upper Pragian of Belgium [39]; upper Lochkovian to lower Emsian of Paraná and Solimoes basins, Brazil [40,41]; Lochkovian to Emsian of Canada [42,43,44]; Lochkovian of Wales [45]; Emsian of Saudi Arabia [46]; and Pragian of France [35].

*Retusotriletes maculatus* occurs from the middle Přídolí to lowermost Eifelian of Libya [31,47]; Pragian to Givetian of Saudi Arabia [36]; Lochkovian of Poland [48]; lower Lochkovian to lower Emsian of Brazil [31,40,41]; Lochkovian to Emsian of Bolivia [49,50] and Canada [44]; Pragian of the UK [51]; and from Pragian to lower Emsian of France [35].

The last Silurian–Devonian TAP miospore is *Brochotriletes tripapillatus* reported from the Givetian of Libya [36] that differs from all previously mentioned species due to the reticulate sculpture of its distal surface.

The stratigraphically oldest record of *Acinosporites lindlarensis* is from the Wenlockian of Argentina [52]; Emsian of Belgium [53], Canada [54], Saudi Arabia [55], Germany [56,57], the UK [58], the USA [59], Tunisia-Libya [60], Czech Republic [61], China [62] and Bolivia [63]; Eifelian of the UK [49], Saudi Arabia [64], Bolivia [65], Poland [66], Canada [67], Germany [56], Tunisia [68], Libya [60], Brazil [69], Czech Republic [61] and China [62]; and Givetian of Canada [70], Czech Republic [61], the USA [71], the UK [72], China [73], Libya [60], Australia [74], Bolivia [68] and Poland [66].

**Figure 2:**
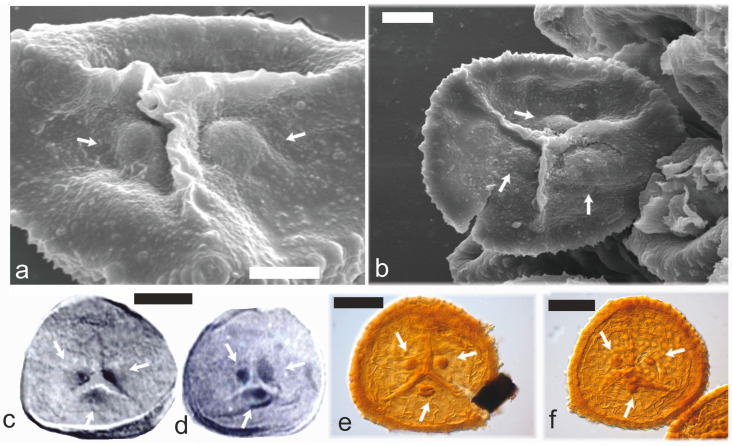
Three apical papillae on some Devonian and Carboniferous microspores. (**a**,**b**) Proximal surfaces of microspores of the *Lundbladispora* type isolated from selaginellalean species *Thomasites serratus*, Ovčín locality, Pennsylvanian (upper Duckmantian) of the Czech Republic. Arrows show two (**a**) and three (**b**) prominent apical papillae. SEM, scale bars 10 µm. (**c**,**d**) Isolated central bodies of microspores of the *Endosporites* type macerated from *Polysporia* sp., Standardsburg, Huron County, upper Devonian (Famennian), USA. Three arrows show three apical papillae. (**c**,**d**) Scale bar 20 µm. (**e**,**f**) Scale bars 15 µm.

Morphologically different Devonian–Carboniferous miospores with TAP are trilete monopseudosaccate species of the genus *Endosporites* where the central body of these spores sometimes possesses TAP [20,75] that can be seen especially if the central body is separated. Globally, the oldest record of in situ *Endosporites* with TAP (Figure 2c,d) is from the upper Devonian (Famennian) of the USA, [20] but the genus is typical for Carboniferous strata.

Carboniferous TAP spores are very rarely reported when compared to those of Silurian/Devonian age. Although trilete cingulate miospores of the genus *Lycospora* belong to the most abundant Paleozoic spores with more than fifty species [76], only one, *Lycospora tripapillata* from the middle Pennsylvanian of USA [77], possesses TAP. Similarly, within another trilete cingulate genus *Densosporites* (one of the most abundant Carboniferous miospores), only one species, *D. tripapillatus* from the Mississippian of the USA, has TAP. The dispersed trilete cingulate miospore genus *Crassispora* has more than 40 species [78] and its first taxa appeared in the Devonian [79,80]. The most abundant species of the genus is *C. kosankei* that is defined without TAP [81,82,83] but sometimes it is described and illustrated with them [77]. Another species, *C. plicata*, is clearly defined and illustrated only with prominent TAP [84]. The Pennsylvanian miospore genus *Thomasospora* with the only species *T. gigantea* (Figure 2a,b,e,f) is also characterized by a distinctive TAP [85], trilete character and equatorial cingulum.

Importantly, we note that all Paleozoic TAP spores are trilete and, except for the monopseudosaccate *Endosporites*, all of them possess equatorial crassitudo/cingulum and have central/inner bodies that bear trilete marks.

### 3.3. Botanical Affinity of TAP Spores

It is possible to determine the botanical affinity of spores and pollen only by their research in situ, i.e., those isolated directly from the reproductive organs of plants. Stratigraphically, the oldest in situ TAP spores are trilete equatorially crassitate isospores of the *Synorisporites papillatus* and *Aneurospora* types isolated from *Cooksonia pertonii* subsp. *apiculispora* from the Lochkovian of UK [11]. TAP spores are known only in few species of the trilete crassitate genera *Ambitisporites, Aneurospora* and *Retusotriletes*, which are produced by rhyniophytoid plants. Another spore genus with TAP is *Brochotriletes* but we still do not know its parent plant.

Another Upper Devonian lycopsid *Cymastrobus irvingii* from the Famennian of Australia [2] yielded trilete in situ microspores with prominent TAP on some probable central bodies but a hypothetical pseudosaccus was not preserved and showed trilete megaspores of the *Valvisisporites auritus* type (without TAP). *Kosoviella timanica* from the upper Devonian of Russia [2] produced TAP trilete cinguate microspores, probably of the *Cristatisporites* type.

Four species of isoetalean lycopsid *Leclercqia* were palynologically studied including *L. andrewsii* from the Emsian of Canada, *L. complexa* also from the Emsian of Canada, *L. uncinata* from the middle Devonian of China and *Leclercqia* sp. from the Emsian of Canada [7]. Although spores from all palynologically studied *Leclercqia* species were compared to the same miospore species, *Acinosporites lindlarensis*, they exhibited a large range of morphological variation. In situ *Leclercqia* spores were compared [7] with dispersed *Acinosporites lindlarensis* Morphon of the late Emsian to the early Givetian. The in situ spores exhibited “palingenesis”; immature spores of *L. complexa* resemble the mature spores of *L.* sp. [7], thus connecting the two known plant/spore forms in the *Leclercqia* lineage. The *Leclercqia* palynodemes exhibited two tendencies with time: (a) curvatural spinae became larger and (b) the proportion of spores with small distal sculptures decreased.

The taxonomic position of the ligulate lycopsid *Leclercqia* is interesting. It is the oldest ligulate lycopsid with a strange mix of characteristics: forking leaves, exarch protostele, pitting of tracheids and presence of ligule. It seems that in *Leclercqia*, various characteristics have been selected by evolutionary pressures at different rates. *Leclercqia* is usually referred to as a Protolepidodendrale, but sometimes [1] is it stated that *Leclercqia* is an intermediate between Lepidodendrales and Protolepidodendrales. Based on palynological data, i.e., in situ spores isolated from four species of *Leclercqia*, we prefer to assign it as a Lepidodendrale than a Protolepidodendrale.

Another isoetalean lycopsid *Oxroadia gracilis* from the Tournaisisan of UK [2] yielded TAP microspores, probably of the *Anapiculatisporites* type, and TAP megaspores belonging to the dispersed megaspore genus *Setispora*. The taxonomic position of *Oxroadia gracilis* is still not quite clear, and it is usually assigned as transition between Protolepidodendrales and Lepidodendrales but based on the occurrence of TAP micro- and megaspores, *Oxroadia* belongs to the Lepidodendralean lycopsids.

Taxonomically and phylogenetically important is the lycopsid isoetalean species *Bisporangiostrobus harissii* from the late Devonian (Famennian) of the USA. Its in situ microspores of the *Geminospora lemurata* type possess prominent TAP; *Bisporangiostrobus* belong to the order Bisporangiostrobaceae [86] and is not a member of rhyniophytes but it belongs to a new TAP isoetalean lineage probably evolved from rhyniophytes within the Emsian. *Bisporangiostrobus harissii* with *Geminospora lemurata* can be the first member of the non-rhyniophytoid TAP isoetalean lineage because all other *Geminospora lemurata* producers appeared later [86] and the first dispersed *G. lemurata* spores appeared within the Emsian [84].

All other Paleozoic in situ TAP spores are from Carboniferous and Permian strata including *Endosporites* microspores isolated from the Stephanian sub-arborescent lycopsid *Polysporia radvanicensis* [75] and *P. doubingeri* [87] and Devonian *Polysporia* sp. [20]. It is important that Famennian *Polysporia* sp. from Ohio, USA [20] yielded not only TAP *Endosporites* microspores but also TAP trilete in situ *Valvisisporites* megaspores that were produced by several species of *Polysporia/Chaloneria* [20]. Sometimes TAP in situ microspores isolated from upper Devonian isoetalean lycopsid *Cymastrobus irvingi* [21] are interpreted as the *Endosporites* type but potential monopseudosaccus was not observed.

In situ trilete cingulate TAP spores of the *Crassispora* type were produced by the arborescent lycopsids *Sigillaria* and *Mazocarpon* (probably a coal-ball counterpart to *Sigillaria*) [88].

TAP cingulate spores were also produced by some herbaceous lycopsids, e.g., *Thomasites serratus* from the upper Duckmantian of the Czech Republic that yielded microspores of the *Thomasospora gigantea* type [85].

Cingulate *Densosporites* spores with TAP were isolated from the herbaceous lycopsid *Porostrobus zeilerii* [89,90] from the Mississippian of Spitzbergen, Norway, and the sub-arborescent species *Omphalophloios feistmanteli* [78] from the upper Duckmantian of the Czech Republic.

TAP miospore species of the cingulate genus *Lycospora* has never been macerated from reproductive organs of parent plants, but we can be sure that *Lycospora* was produced only by arborescent lycopsids of the *Lepidodendron* type that produced cones of the *Lepidostrobus* type because we do not know another its producer and the *Densosporites* miospores are produced by sub-arborescent lycopsids of the *Omphalophloios* and herbaceous lycopsids of the *Selaginella* type [7].

Another in situ record [24] is the trilete cavate Permian miospores species *Densoisporites polaznaensis* with prominent TAP and the associated isoetalean lycopsid genus *Viatcheslavia vorcutensis* from the Guadalupian of Russia. Three species of the Mesozoic isoetalean lycopsid plant genus *Pleuromeia, P.* sp. from the Triassic of Romania, *P. rossica* from the Triassic of Russia and *P. sternbergii* from the Triassic of Germany [18,19] were palynologically studied for in situ spores. *Pleuromeia* sp. yielded TAP megaspores of the *Talchirella daciae* type [18], *P. sternbergii* produced TAP microspores of the *Densoisporites nejburgii* type and TAP megaspores *Trileites polonicus* [18,19] and *P. rossica* produced in situ microspores assigned to *Densoisporites neuburgae* from the Mesozoic (Triassic) *Pleuromeia rossica* and [22] also megaspores of the *Trileites polonicus* type. TAP are not visible on these megaspore surfaces using LM microscopy but they were clearly seen when looking at the central body using SEM. Interestingly there are not only three papillae but several papillae (a few hundreds) along the rays of the trilete mark; TEM analysis of these megaspores shows the occurrence of LZs.

The isoetalean lycopsid *Annalepis zeilerii* from the Triassic of France yielded [23] TAP microspores of the *Aratrisporites saturni* type and TAP megaspores *Tenellisporites marcinkiewiczae*.

TAP spores continued through to the Mesozoic and Cenozoic. The occurrence of several isoetalean taxa from the Mesozoic and Cenozoic has been reported [91], sometimes with in situ spores but without a description of TAP spores probably because TEM was not used. The Mesozoic taxa include species such as *Skilliostrobus australis, Annalepis latiloba, Cylostrobus ornatus, Isoetes circularis, Nathorstiana arborea,* and *Isoetites insignis*; Cenozoic species include *Isoetes reticulata*.

It is important that not only microspores but also some in situ megaspores have laminated zones. Several sculpture elements along the rays of the trilete mark of in situ megaspores of the *Trileites polonensis* type were isolated [23] from the Triassic isoetalean lycopsid *Pleuromeia sternbergii*. These sculpture elements are laminated zones that were recognised by TEM. The same pattern is possessed by Carboniferous microspores of the *Anapiculatisporites* type that was produced by *Carinostrobus foresmani* [90], a member of the selaginellalean clade and probably by [21] *Oxroadia gracilis*.

Not only mio- and microspores possess TAP; we also have some records of TAP in megaspores. The Mesozoic isoetalean lycopsid *Lepacyclotes* (*Annalepis*) *zeilerii* (Triassic of France [23] yielded TAP trilete megaspores of the *Tenellisporites marcinkiewiczae* and monolete microspores of the *Aratrisporites saturni* type. It is interesting that these in situ microspores are not trilete but monolete and possess a central body and monopseudosaccus-like trilete isoetalean microspores of the *Endosporites* type.

Other in situ TAP isoetalean megaspores are known from *Pleuromeia* sp. [21,92] that can be assigned to the dispersed megaspore species *Talchirella daciae* and *Pleuromeia sternbergii* with TAP megaspores of the *Trileites polonicus* type.

### 3.4. New Approach to Phylogeny of Paleozoic Isoetalean Lycopsids Based on Palynological Evidence

**Figure 3:**
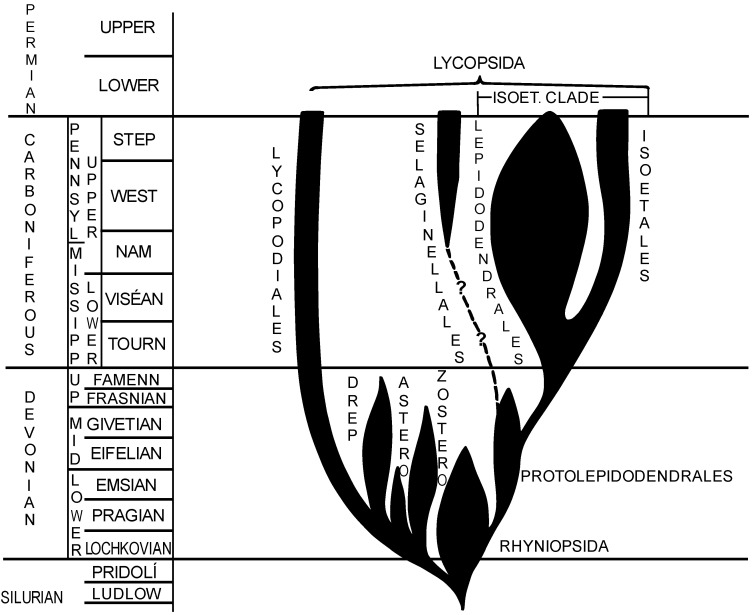
Phylogeny of isoetalean lycopsids, modified from [1].

The generally accepted scheme of phylogeny of Paleozoic isoetalean lycopsids [1] is shown in Figure 3. It suggests that within the Ludlow–Přídolí interval, two main phylogenetic lineages evolved: rhyniophytoids and lycopodialean plants. From the lycopodialean lineage a few lycopsid groups originated including zosterophylls (from the Lochkovian to Givetian), asteroxylaleans (from the Lochkovian to Emsian) and drepanophycaleans (from the Pragian to Famennian). An independent main lycopodialean lineage continues from the Přídolí to today.

It was supposed [1] that two significant lineages, selaginellalean and isoetalean, originated from the group of protolepidodendralean lycopsids. However, the palynological problem of this scheme is that TAP spores occurring in some rhyniophytoids were never been recorded in any member of the protolepidodendraleans., i.e., TAP spores would have had to disappear (“hiatus”) and appear again in spores of the isoetalean clade some tens of millions of years later. This seems less probable.

**Figure 4:**
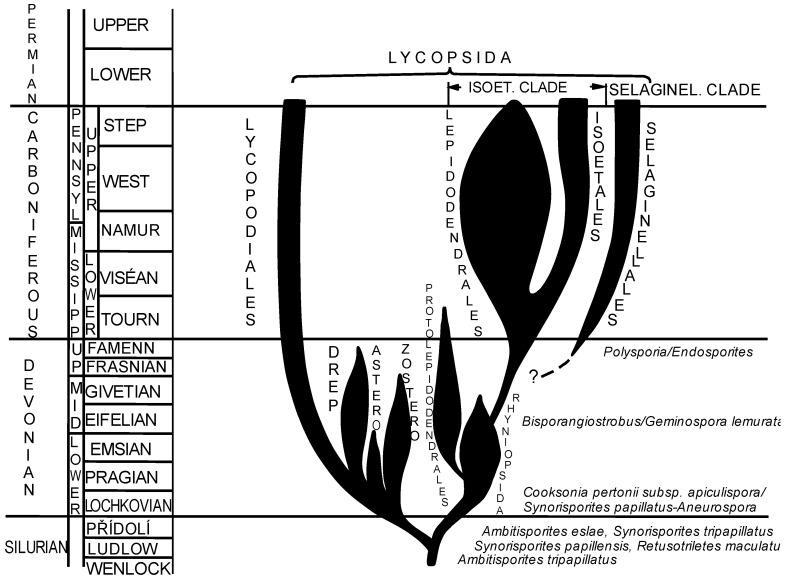
New scheme of phylogeny of isoetalean lycopsids.

It is possible to postulate a new hypothesis supported by the palynological data and by the uninterrupted continuation of a TAP line from the lower Silurian (Wenlockian) to Cenozoic. If TAP spores are included into this scheme, we can see a different hypothetical phylogenetic scheme of Paleozoic lycopsids, especially the isoetalean and selaginellalen clades (Figure 4). Protolepidodendrales could evolve from rhyniophytoids within the Lochkovian as a “blind” line disappearing in the Tournaisian (Mississippian, i.e., lower Carboniferous) and not in the Frasnian (Devonian) as assumed [1]. The TAP line could originate in the Wenlockian within rhyniophytes because the first TAP spores (*Ambitisporites triapillatus*) were described here for the first time. TAP spores are reported continuously from the Wenlockian to Givetian within rhyniophytes and from the Devonian (Emsian), Carboniferous and continuing to the Mesozoic and Cenozoic.

Some spores of plants of the selaginellalean clade also possess TAP, i.e., they probably did not evolve from protolepidodendraleans (that lack TAP spores) as believed [1] but from a TAP-independent lineage probably within the middle/late Devonian.

## 4. Conclusions

We can understand isoetalean lycopsids producing TAP spores as an independent phylogenetic lineage. The principle of the TAP hypothesis is that this lineage continued from the Wenlockian (lower Silurian) through the Devonian, Carboniferous, Permian, Mesozoic and Cenozoic as documented by dispersed and in situ TAP spores. The TAP lycopsid lineage consists of the selaginellalean and isoetalean (isoetales and lepidodendrales) clades that did not evolve from protolepidodendraleans but directly from rhyniophytoid plants. This hypothetical concept reflects the palynological data that were not included into previous phylogenetic schemes. Based on the palynological data, we assigned the lycopsid genera *Leclerquia* and *Oxrodia* to Lepidodendrales and not to Protolepidodendralean plants.

It seems that Paleozoic TAP isoetalean microspores should be divided into two groups. The first group consists of Silurian–Carboniferous trilete TAP spores of some species of the genera *Ambitisporites, Synorisporites, Streelispora, Brochotriletes, Thomasospora, Crassispora, Lycospora, Densosporites* and *Cristatisporites*. The second group are some Devonian–Carboniferous trilete TAP cavate/pseudosaccate species of the genera *Acinosporites, Geminospora* and *Endosporites*. Mesozoic isoetalean TAP spores are morphologically similar. TAP/LZs are usually three verrucae/granae close to the proximal pole between the rays of the trilete mark but their number can be higher (some tens or even a few hundreds).

## Figures and Tables

**Table 1:** List of rhyniophytoid plants producing spores from the genera *Ambitisporites*, *Synorisporites*, *Retusotriletes* and *Aneurospora*.

Parent Plant	In Situ Spores	Stratigraphy	References
*Cooksonia pertoni* subsp. *pertoni*	*Ambitisporites*	Lochkovian	[10]
*C. pertoni* subsp. *synorispora*	*Synorisporites verrucatus*	Přídolí	[10]
*C. pertoni* subsp. *apiculispora*	*Streelispora newportensis/Aneurospora*	Lochkovian	[11]
*Caia langii*	*Retusotriletes*	Přídolí	[11]
*Cooksonia cambrensis*	*Ambitisporites*	Přídolí	[12]
*Pertonella dactylethra*	*Retusotriletes coronatus*	Přídolí	[12]
*Renalia hueberi*	*Retusotriletes/Apiculiretusispora*	Lochkovian	[13]
*Salopella allenii*	*Retusotriletes*	Přídolí	[14]

## Data Availability

The data presented in this study are available on request from the corresponding author.
